# Femtosecond laser induced step-like structures inside transparent hydrogel due to laser induced threshold reduction

**DOI:** 10.1371/journal.pone.0222293

**Published:** 2019-09-17

**Authors:** Emanuel Saerchen, Susann Liedtke-Gruener, Maximilian Kopp, Alexander Heisterkamp, Holger Lubatschowski, Tammo Ripken

**Affiliations:** 1 Laser Zentrum Hannover e.V., Hannover, Germany; 2 Rowiak GmbH, Hannover, Germany; 3 Institut fuer Quantenoptik, Leibniz Universitaet Hannover, Hannover, Germany; Nicolaus Copernicus University, POLAND

## Abstract

In the area of laser material processing, versatile applications for cutting glasses and transparent polymers exist. However, parasitic effects such as the creation of step-like structures appear when laser cutting inside a transparent material. To date, these structures were only described empirically. This work establishes the physical and chemical mechanisms behind the observed effects and describes the influence of process and material parameters onto the creation of step-like structures in hydrogel, Dihydroxyethylmethacrylat (HEMA). By focusing laser pulses in HEMA, reduced pulse separation distance below 50 nm and rise in pulse energy enhances the creation of unintended step-like structures. Spatial resolved Raman-spectroscopy was used to measure the laser induced chemical modification, which results into a reduced breakdown threshold. The reduction in threshold influences the position of optical breakdown for the succeeding laser pulses and consequently leads to the step-like structures. Additionally, the experimental findings were supplemented with numerical simulations of the influence of reduced damage threshold onto the position of optical breakdown.

In summary, chemical material change was defined as cause of the step-like structures. Furthermore, the parameters to avoid these structures were identified.

## Introduction

Ultrashort laser pulses are used for precise, three dimensional applications under the surface of optical transparent materials in laser-material processing [1–3] and laser medicine [4–5]. For these purposes, lasers with pulse durations of several hundreds of femtoseconds (fs) and low pulse energies combined with high pulse peak powers are applied. The localized disruption inside the material is achieved by tight focusing of the laser light, while inducing low thermalisation. Areas axially above and below the optical focus remain apparently unaffected. This results in precise manufacturing of the material with micrometer-(μm) and sub-micrometer resolution [6–8].

The cutting effect is achieved by femtosecond-laser induced photodisruption. This process is well understood for single pulses [9]. If the damage threshold of the material (approx. 10^12^ W/cm^2^ [10, 11]) is reached by tight spatial focusing of the laser pulse, the critical electron density of 10^21^ cm^-3^ [11, 12] is achieved. Herewith, the bonding energy of the electrons to the nucleus will be exceeded, which results in an ionization processes due to multiphoton-, avalanche- or tunnel-ionization [13]. The plasma persists for some picoseconds until recombination takes place [9]. During the relaxation processes, the electron energy is converted into heat followed by a generated shock wave due to the high temperature discontinuity in the focal region [9]. A high water content of the material leads to an expanding and collapsing gas bubble [14]. In our experiments, a gas bubble has not been observed due to a low water content. Hence, the cutting effect was more dominated by plasma generation.

With this processing technique, for example nano-gratings for polarizers, phase masks or fiber Bragg gratings can be produced [2, 15]. Laser-processing inside transparent dielectrics shows additional high potential for the creation of integrated optical circuits as interferometers or diffraction gratings for spectral analysis [16]. The aim of this development is to fabricate ‘circuit on a chip’ with optical switches, mirrors and beam splitters on one single substrate [17, 18]. Additionally, fs-laser pulses can be used to write optical waveguides by inducing refractive index changes inside of the material [19, 20]. In waveguide writing, the effect of heat accumulation by using repetition rates above 100 kHz was observed [21]. This paper will also investigate the influence of pulse repetition rate on the creation of step-like structures. In biomedicine, the fs-laser is used for processing of intra-ocular lenses (IOL). Herewith, the laser is used for cutting [22] as well as creating refractive [23] and diffractive optical structures [24] inside the IOL. Thus, an additional or individualized refraction power of the IOL is achieved to obtain the correction of ametropia. Besides, the fs-laser can be used for histological thin sectioning of hard or brittle organic samples [2, 25].

For all those applications, the precision of cuts is of vital importance [26] as well as shortening processing durations. Consequently, the trend goes to lower pulse energies of several nano-Joules and high repetition rates in the MHz-range [20, 27]. Although relevant for a successful application, not all interaction processes between laser and material are adequately described and understood yet [14].

**Fig 1:**
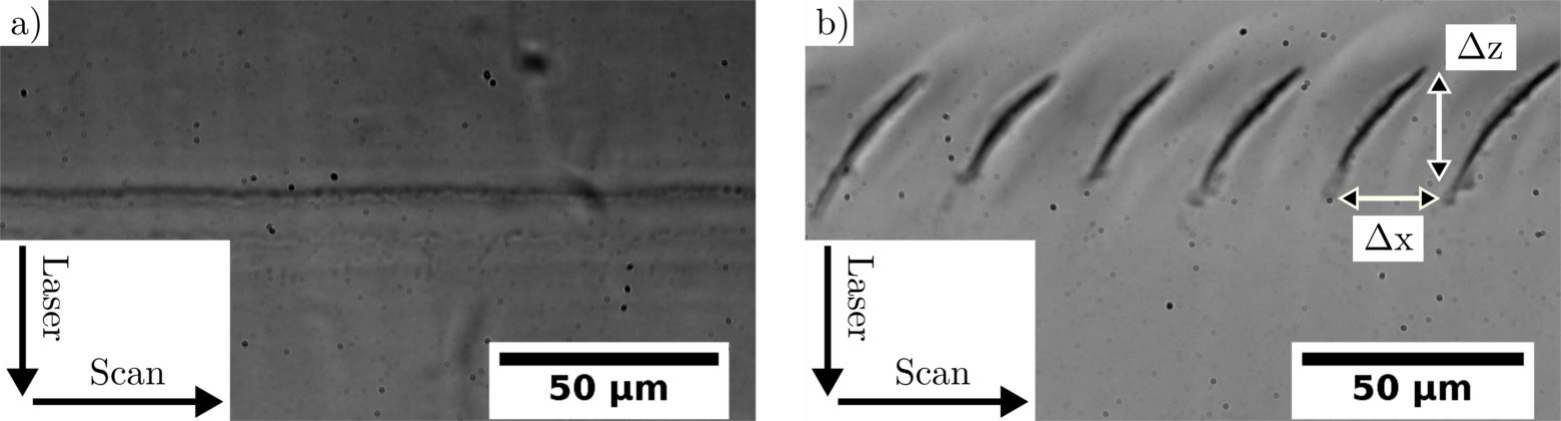
Side view of optical light microscope image of laser applied lines in transparent material HEMA with spatial pulse distance of 10 nm (a) and 0.1 nm (b). The laser was focused with numerical aperture 0.6 and pulse energy 210 nJ from top to bottom inside a depth of 300 μm in the sample material. The lines were cut from left to right. Image b) shows the creation of step-like structures. Each step is due to 200,000 laser pulses.

Cutting lines or planes inside the transparent material represents a typical application in laser-material processing [2, 22, 28]. Therefore, several pulses are applied next to each other. The destruction caused by one single fs-pulse overlaps with the destructive region of neighboring pulses, which causes the formation of a line. Several lines next to each other result in planes. However, using specific processing parameters leads to the creation of unintended step-like structures, which are the topic of this paper. Fig 1B shows such a laser cut line inside the transparent material from side view. The laser was focused from top inside the material. The sample was moved laterally to position several pulses next to each other. The spatial pulse distance was varied between Fig 1B and Fig 1A. All other parameters as pulse energy, numerical aperture, pulse repetition rate and the sample material were kept constant. The profile of the laser application shows a step-like structure. Step height Δz is defined as the axial size of one step, whereas step width Δx is defined as the lateral size of one step (see Fig 1). The steps are the results of several thousand laser pulses. The step height can become ten times the Rayleigh length.

This effect has already been observed so far in hydrogels as Dihydroxyethylmethacrylat (HEMA) [29–31] and polycarbonate (PC) [32, 33]. The work of Schumacher and Shaltev reveals that step-like structures with step-heights of 102 μm can be achieved in HEMA by fs-laser application with 1.4 μJ pulse energy, 2 μm spatial pulse separation and 0.18 numerical aperture at 100 kHz repetition rate [30–31]. The step height increases as the spatial pulse distance is lowered. A relation to pulse energy and temporal pulse separation was not investigated. Vartapetov *et al*. report on step heights of 104 μm in polycarbonate by using 150 nJ pulse energy, 2 kHz repetition rate, 20 nm spatial pulse distance and numerical aperture of 0.39 [33]. The step height was as well inverse proportional to spatial pulse distance [33]. The explanation attempt was a laser induced change in refractive index, which results in an additional focusing effect of the laser beam [32, 33]. Additionally, a variation of the density and structure of the material should have reduced the threshold for optical breakdown and led to the step-like structures [32, 33]. However, the authors have not done further investigation to justify those hypothesis.

Likewise, in fused silica [34–38] the creation of periodic structures was investigated as well, which show similarities to the effect of this paper. Widening of the periodic structure was related to an increase in pulse energy and repetition rate [37] as well as enlarged spatial separation distance of the pulses [34]. The measured period of approx. 40 μm [34] is comparable to the step widths of the following study. A pulse energy of 300 nJ, repetition rate of 9.4 MHz and numerical aperture of 0.3 was used therefore [34]. Additionally, an influence of the laser polarization onto the orientation of the periodic structures was observed [35]. As possible reason of the effect in fused silica, an increase in density due to multiple pulses was assumed [36, 39]. Moreover, the laser induced melting and uneven solidification of the glass could have caused refractive index variations which resulted in additional aberrations and consequently a reduction of laser-intensity below the threshold [37, 40]. This resulted in a periodic cutting of the material [37, 40].

To sum up, different hypothesis about the cause of the creation of step-like structures were postulated, but not enough evidence for the different theories was compiled. For a successful fs-laser processing, the physical cause of this effect needs to be identified and laser parameters needs to be defined, to suppress the creation of step-like structures.

## Material and methods

The experiments were performed by using the TissueSurgeon (ROWIAK LLS GmbH, Germany) [41], containing a t-pulse 500 femtosecond laser (Amplitude Systèmes, France) at 1030 nm wavelength, with 350 fs pulse duration, 10 MHz repetition rate and 500 nJ pulse energy. The pulse energy was varied by using a half-wave plate and a polarization dependent beam splitter cube. Laser light was focused with a custom made objective lens of 0.6 numerical aperture inside the material. This led to a diffraction limited spot radius ω_0_ of 0.8 μm and a Rayleigh range z_r_ of 2.7 μm. The repetition rate was varied from single pulses up to 10 MHz. The objective lens was manufactured by a commercial optic company and optimized to obtain the smallest spot size for 1030 nm wavelength at a depth of 70 μm in the sample material after passing a glass object slide with 1.0 mm thickness. The sample material was the polymer Dihydroxyethylmethacrylat (HEMA from Contamac Ltd., United Kingdom [42]), which was used with a constant water content of 38%. Because the polymer usually serves as material for contact and intraocular lenses [43, 44], this polymer was used representatively for ocular tissue material to link the results of the laser-material interaction to the laser-tissue interaction for ocular laser treatments. The physical and chemical properties of hydrated HEMA are constant for several months. The chemical formula of HEMA is C_6_H_10_O_3_. The polymer consists of one OH-group, one C = C- group and one C = O-group. For the duration of the laser treatment, the polymer was stored constantly in distilled water. During the experiments the sample was placed on a 1 mm thick object slide (Carl Roth GmbH, Germany). Furthermore, a chamber was attached to the object slide, which was filled with water. Herewith, a dry out of the samples was prevented. The object slide was moved via x-, y-, z- translation stage (KDT105 Steinmeyer, Feinmess Dresden GmbH, Germany). The desired cutting depth was addressed by using an integrated OCT-imaging technique simultaneous to the cutting process.

To investigate the morphology of the laser-induced step-like structures, lines with different pulse energies, as well as spatial and temporal pulse distance were cut inside the material. After processing the sample, an inverse microscope (Axio Observer. D1, Carl Zeiss AG, Germany) with high resolving objective (N-Achroplan 100x/ /1.25 Oil, Carl Zeiss AG, Germany) was used to investigate the structures in top- and side- view geometry. For side-view, the sample material was cut in half by a razor blade.

In addition, samples were analyzed by confocal Raman-microscopy (CRM200, WITec GmbH, Germany) from *Hannoversche Zentrum für optische Technologien (HOT)* to obtain information about the chemical decomposition after the laser treatment [45, 46]. Excitation of the sample was done by frequency doubled Nd:YAG laser at 531.9 nm. Signal detection of the Raman-scattering was done by spectrometer (UHTS 300, WITec GmbH, Germany) and electron multiplying charge-coupled device (emCCD) camera (DU970N-BV-353, Andor Technology Ltd., United Kingdom). The spectral measurement range was from -80 to 3710 rel. cm^-1^ [46]. This corresponds to 530–663 nm. Imaging of the samples was done by 60 x objective (CFI Fluor 60x, NA 1.0, Nikon Corp., Japan).

## Results

The damage threshold at 70 μm depth was measured to be at 80 ± 5 nJ. This was the smallest measured pulse energy to reach the damage threshold, which confirms the designed focal depth of the used objective lens. By applying single pulses inside HEMA in a depth of 70 μm below the sample surface, a disrupted cone of material with the size of 6 ± 1 μm axial and 2 ± 1 μm lateral at maximum pulse energy of 230 nJ was measured. Hence, if several pulses were placed next to each other to cut a line inside the material, the axial size of the structure should not exceed 6 μm. However, apparently a much larger size up to 40 μm was addressed (see Fig 1B), due to the interaction of multiple laser pulses. Applying several pulses in a line next to each other resulted either in a well aligned line or in the creation of step-like structures.

**Fig 2:**
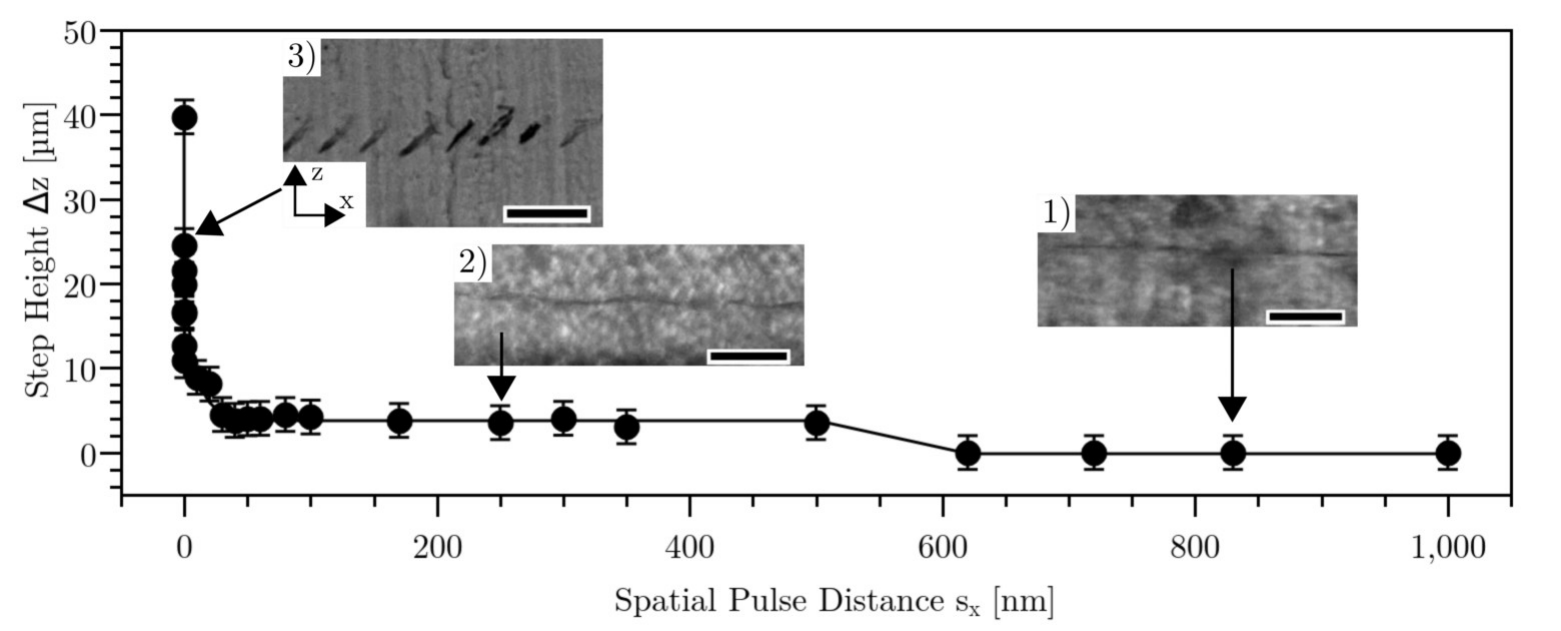
Axial step height Δz vs. spatial pulse separation distance sx for constant pulse energy of 236 nJ. The measurement points were connected for illustration purposes. The error bars derive from the standard deviation over 11 values. The measurement points below 0.16 nm are shown in Fig 3. Inlet: Bright field images show representative structures in side view: (1) straight laser cut line, (2) wave-like laser cut line, (3) step-like laser cut line. Scale bar length is 50 μm.

**Fig 3:**
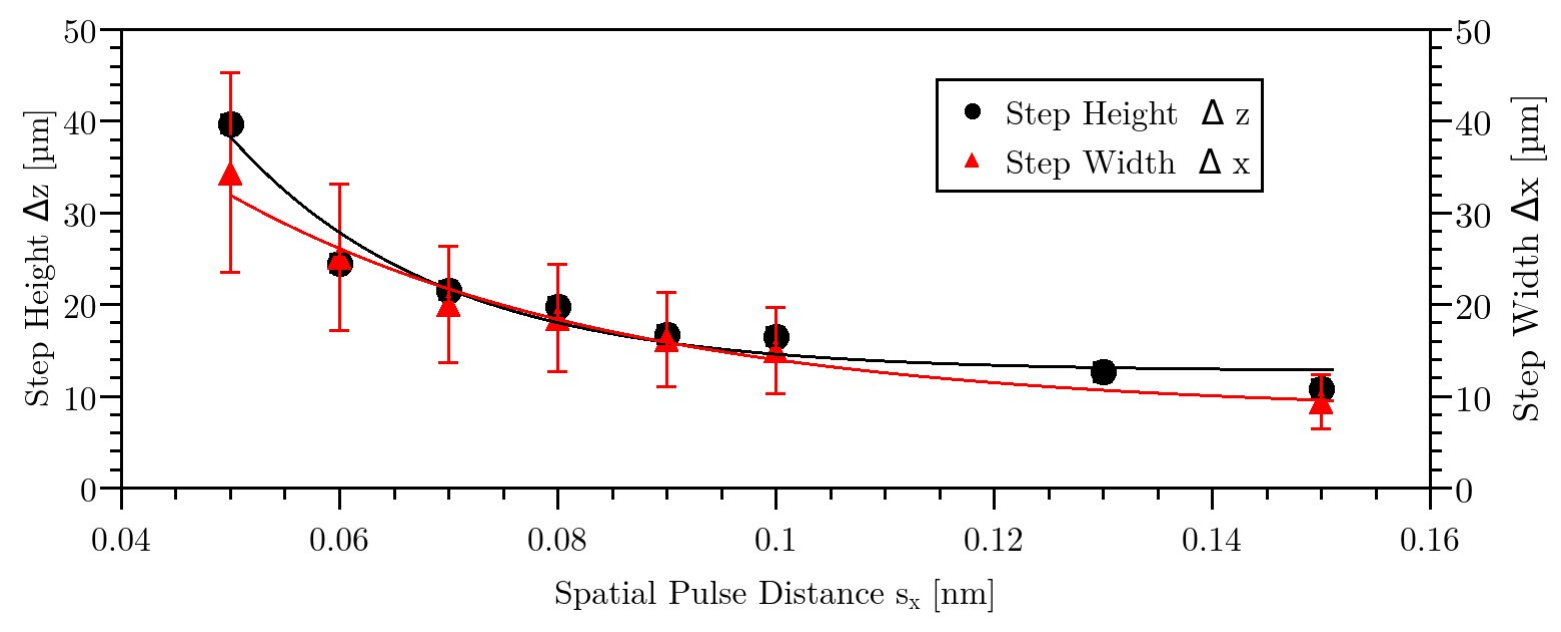
Axial step height Δz and step width Δx as a function of spatial pulse separation distance s_x_ for 236 nJ pulse energy. The error bars derive from the standard deviation over 11 values.

For the following investigation, lines with different lateral laser pulse distances s_x_ were applied inside the material. By variation of laser repetition rate and speed of the stage, a variation of pulse distance s_x_ from 0.05 nm (99.996% pulse overlap) to 1 μm (31% pulse overlap) was achieved. Varying the repetition rate also effected the time between the applied pulses. This will be described further down. A constant pulse energy of 236 nJ was used. Varying the pulse distance resulted in step-like structures with different height Δz and width Δx of the steps (see Figs 2 and 3). Furthermore, representative bright field images of the step-like structures in side view were obtained. For pulse distances below 0.16 nm, the trend of the curve was exponentially (see Fig 3). The value for step height and step width was an average of 11 measured steps with standard deviation for each corresponding pulse distance.

Fig 2 indicates that there are three regimes of the step height Δz while the pulse distance was varied. The first regime was at a pulse distance s_x_ > 0.6 μm (< 59% pulse overlap). For this regime, the step height Δz could not be measured (see Fig 2). For the second regime, at pulse distances between 50 nm (97% pulse overlap) and 0.5 μm (66% pulse overlap), a slightly periodic behavior of the applied line inside the material was visible, with a step height of ca. 4 μm (Fig 2). For the third regime, below 50 nm pulse distance, a distinct periodic structure caused by laser-material-interaction was visible (see Fig 2). In this regime, a maximal step height Δz of 40 μm was measured. The measured data were linked with a Gaussian fit with R^2^ = 0.95. The step width shown in Fig 3 applies only for the third regime, because in the other regimes the line was not separated so that the step widths could not be determined. In this figure, the measured data were linked with a Gaussian fit with R^2^ = 0.98. Step height and step width behave directly proportional to each other.

**Fig 4:**
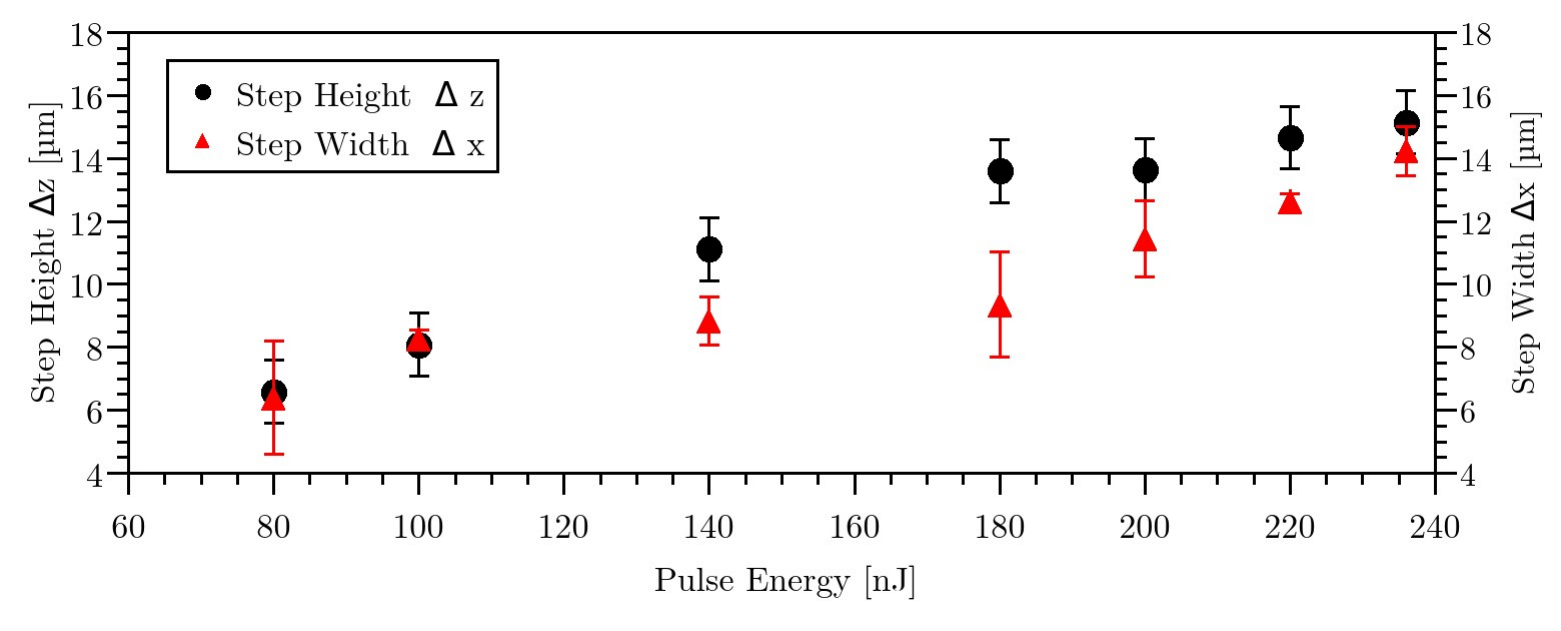
Step height and step width as a function of laser pulse energy for constant spatial pulse separation distance of sx = 0.13 nm. The error bars derive from the standard deviation over 3 values.

Additionally, the influence of laser pulse energy on the step height Δz and step width Δx was investigated. Therefore, the lateral pulse distance s_x_ was kept constant at 0.13 nm (99.991% pulse overlap). Lines were cut in a depth of 70 μm inside HEMA. For each applied pulse energy, an average and standard deviation of three measured step heights and step widths were obtained, as depicted in Fig 4. The step width increases from 6.4 μm at 80 nJ to 14.2 μm at 236 nJ. A similar behavior could be seen for the step height, which increases from 6.6 μm to 15.1 μm, respectively. The step width was again proportional to the step height.

**Fig 5:**
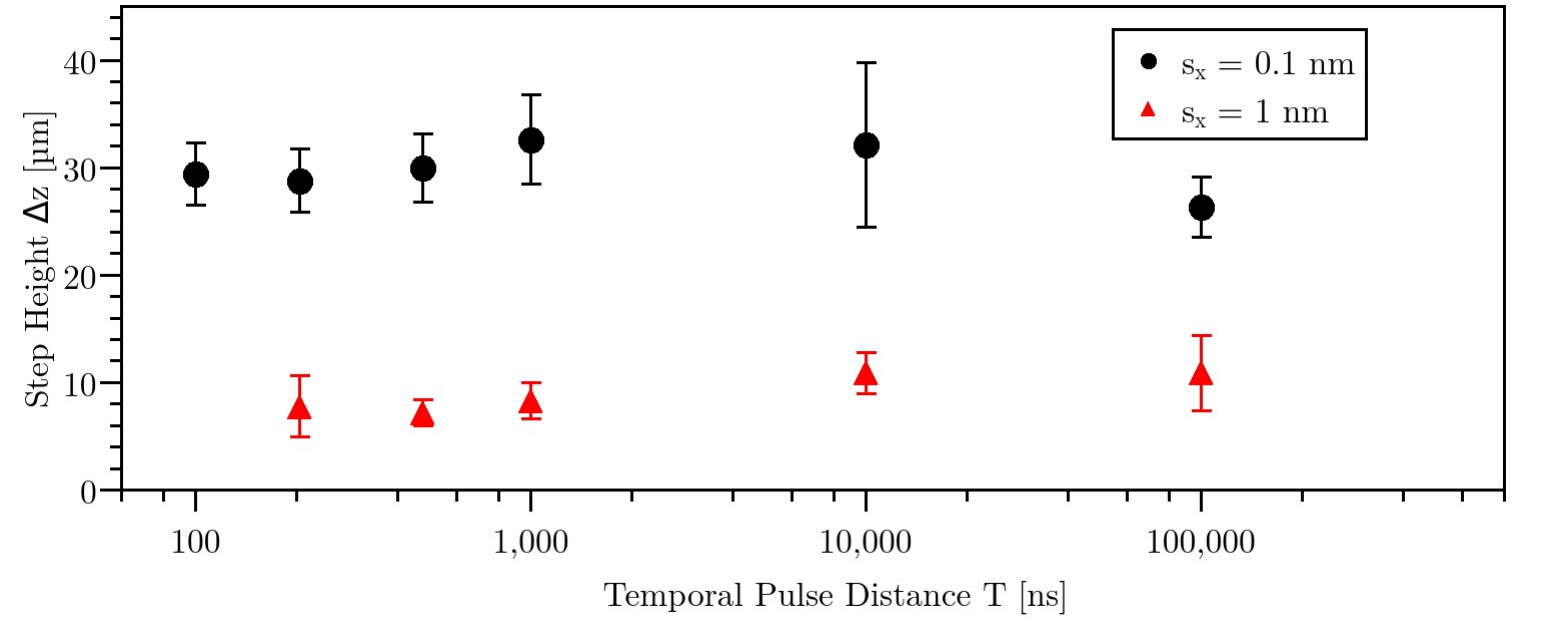
Step height as a function of temporal pulse distance T for two different spatial pulse separation distances sx at 210 nJ pulse energy. The error bars derive from the standard deviation over 10 values.

The previously investigated spatial pulse distance was obtained by the movable stage and the variation of the laser pulse repetition rate. Notice that changing the repetition rate also affected the temporal pulse distance, and, accordingly, the step height of the previously described step-like structure. Furthermore, an influence of the temporal pulse distance on the step height would indicate a thermal cause of this effect. To investigate this, a variation of repetition rate between 10 kHz and 10 MHz was used for either a constant spatial pulse distance of 0.1 nm (99.993% pulse overlap) or 1 nm (99.93% pulse overlap). The cutting depth was 300 μm with pulse energy of 210 nJ (see Fig 5). Shown are averages and standard deviation of 10 measured step-like structures.

For repetition rate between 10 kHz and 10 MHz, significant dependence of step height on temporal pulse distance cannot be seen. This indicates that the formation of the step-like structures may not be attributed to heat accumulating effects. In other transparent dielectrics, heat accumulating effects were measured above 100 kHz repetition rate [21]. Since no influence of temporal pulse distance on step height was seen in the present study, the preliminary investigation method of using different repetition rates and speeds of the linear stage to achieve a variation in spatial pulse separation distance was proven to be correct.

**Fig 6:**
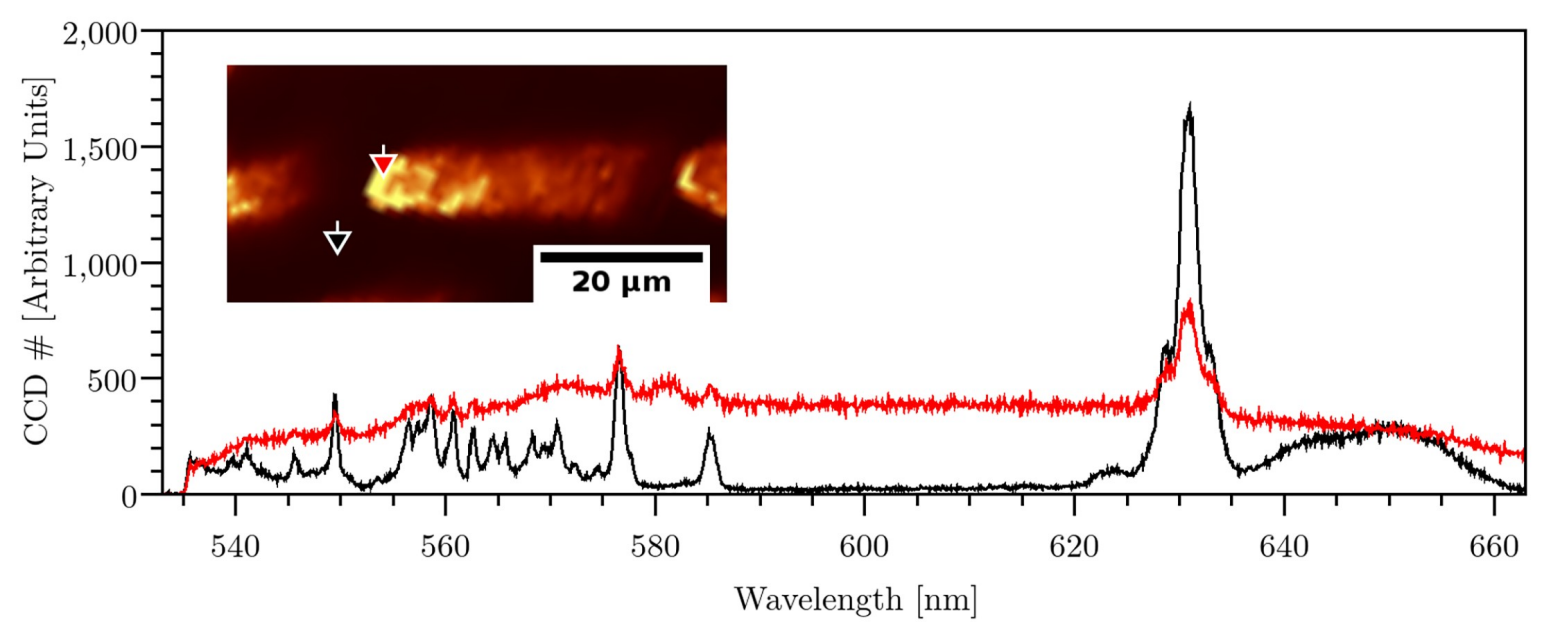
Inlet: Raman-microscopic image of laser structured HEMA. The image shows the average of the spectral intensity values. A step-like structure can be seen from top view, which was created with sx = 0.1 nm and E = 200 nJ. The diagram shows the Raman-spectra (in black and red line) for the positions, which are indicated with red and black arrow, respectively.

Besides the temporal spot separation as an indicator for thermal dependency, the chemical modification of the material caused by laser radiation was analyzed. For this purpose, a step-like structure was produced by focusing laser pulses in a depth of 70 μm with pulse energy of 200 nJ and spatial pulse distance of 0.1 nm. Afterwards, the structure was imaged by Raman-microscopy (see Fig 6). One representative spectra of the laser-modified (red curve) and unmodified area (black curve) was depicted as well. The black curve exhibits characteristic modes of vibration states. The peak at 576 nm (1460 rel. cm^-1^) corresponds to the C − H bending. The peaks between 628–633 nm (2890–3000 rel. cm^-1^) stand for C − H stretching [47]. The peak at 585 nm (1706 rel. cm^-1^) is caused by C = O stretching. Those peaks can also be obtained at polymers Polymethylmethacrylat (PMMA) and Polyethylenterephthalat (PET) [47].

In contrast, the spectrum of the modified material (red curve) are damped, meaning that the relative amplitude of the characteristic peaks was reduced. Thus, some peaks were not distinguishable from the background anymore. The material was strongly dissociated that several vibration states were excited and the abundance of characteristic peaks was relatively reduced. According to [29], this broad fluorescence signal can be explained by the dissociation of polymers in monomers and fluorescent poly-cyclic aromatics. Furthermore, an additional peak between 580 and 583 nm (1560–1650 rel. cm^-1^) appeared, which was not visible in the unmodified area. This additional peak could be explained as C = C stretching [29, 47]. In particular, the polymer HEMA consists of one C = C -group, whose bending could occur with more abundance after the laser-induced dissociation of the polymer. This could be explained by the creation of new molecular groups from the polymer.

In conclusion, the laser-material interaction induces a dissociation of the polymer in other molecular groups, which is an evidence for a reduction of the threshold for optical breakdown.

## Discussion

The experimental results showed a dependency of step height and step width on spatial pulse separation and laser pulse energy, but there is apparently no influence in temporal distance of each single pulses. Applying laser pulses in a line resulted in three regimes for different pulse separation distances. Reducing the pulse distance led to an increase in step height and step width. Both, step height and step width corresponded directly linear to each other. The pulse energy had a linear dependence on step height.

An influence of laser pulse energy and spatial distance on the creation of step-like structures indicated that an intensity dependent laser-material interaction causes this effect. Therefore, the effect of an intensity dependent reduction of the materials damage threshold on the location of the laser-induced optical breakdown for multiple pulses was modeled by using MATLAB (The MathWorks, Inc., USA). The model described a two dimensional room (x, z). At the beginning of the simulation, a constant threshold intensity I_s_(x, z) of 10^12^ W/cm^2^ [10, 11] was apparent. During the simulation, a Gaussian intensity profile of the form

$$I(x,z,N,s_{x})=I_{max}\frac{e^{-2{\left(x-Ns_{x}\right)}^{2}}}{\omega^{2}(z)}$$ (1)

was applied in the medium. Herewith s_**x**_ is the previously described spatial pulse separation distance and N a variable for the number of applied laser pulses. The maximum intensity I_max_ in the center of the laser beam is defined by

$$I_{max}=\frac{A_{b}E_{p}}{\tau \pi \omega^{2}(z)}.$$ (2)

For the following model, the theoretical values should be compared to the experimental data of step height and step width versus spatial pulse separation distance (see Fig 3). Therefore, the pulse energy is E_P_ = 236 nJ, the pulse duration is τ = 350 fs and the energy absorption A_b_ to reach the damage threshold is 30% [10, 48]. The beam radius ω, where the intensity is decreased to the 1 over e^2^ value, is related to the propagation of Gaussian-beams with

$$\omega (z)=\omega_{0}\sqrt{1+\frac{z^{2}}{z_{r}^{2}}}.$$ (3)

The beam radius at the focal point ω_0_ is 0.8 μm and the corresponding Rayleigh-range is z_r_ = 2.7 μm. While the laser beam was focused inside the modeled area, the laser intensity was compared to the threshold for optical breakdown. If the threshold was reached, the propagation of the laser beam stopped and the creation of an optical breakdown was expected to occur. Thereafter, the neighboring laser pulse at distance s_**x**_ propagated into the material. This beam also propagated into the depth, where the damage threshold was reached.

Effects of the optical breakdown as photodisruption with shock wave and cavitation bubble expansion were not taken into account. However, during propagation of the laser beam into the model material, the local threshold value of the material was reduced with respect to the laser intensity. This variation of the material property affected the position where following laser pulses reached the damage threshold. Hence, the laser-affected damage threshold of the material I_s_(x, z, N) is described with

$$I_{s}(x,z,N,s_{x})=I_{s}(x,z,N-1,s_{x})-0.00009I(x,z,N,s_{x})$$ (4)

including the already stated intensity profile of the laser pulse I(x, z, N) of formula 1. The prefactor of 0.00009 was determined iteratively. This factor defines the decrease of damage threshold per pulse.

The model was used to show the effect of different lateral spot distances on the creation and size of step-like structures. The effects of self-focusing were not considered in this model. Self-focusing is negligible for high numerical apertures and affects each applied pulse similarly. Thus, no difference of the results of this modeling without self-focusing was expected.

**Fig 7:**
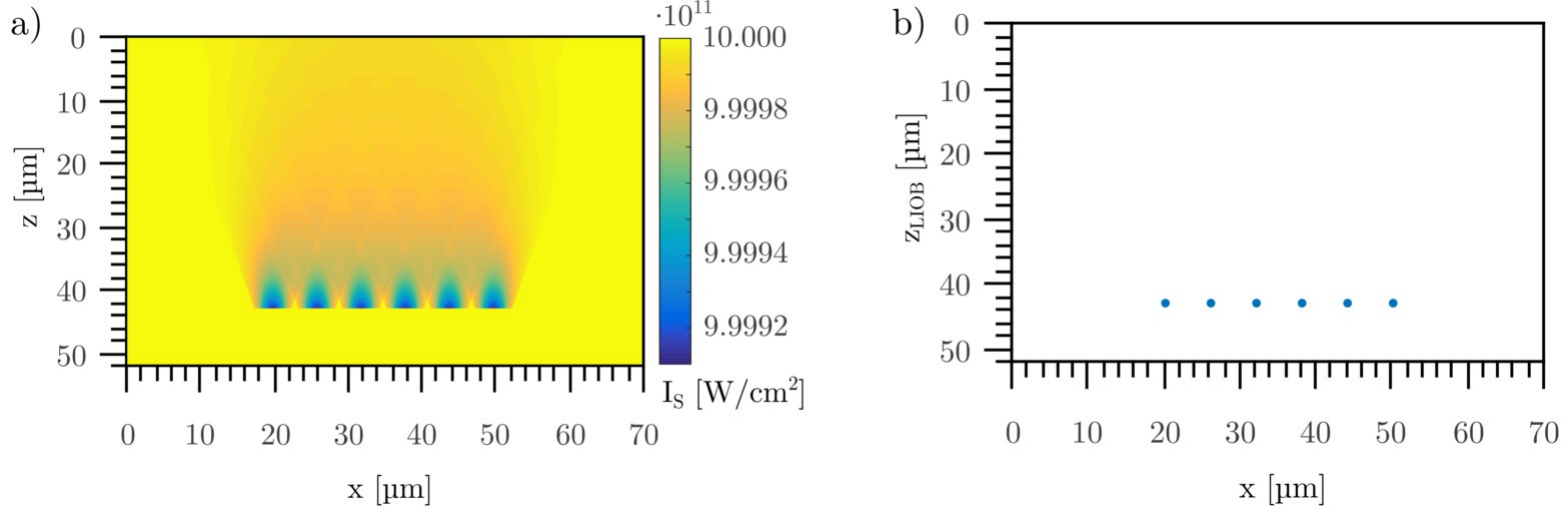
Simulation of six applied pulses with spatial separation distance sx of 6 μm inside a material with intensity dependent variation of the laser damage threshold Is. Shown is the varied threshold Is(x,z) (a) and the position of optical breakdown zLIOB(x,z) (b).

For s_**x**_ = 6 μm, no interaction between the laser induced material modification and the position of achieved damage threshold of the following pulses took place (see Fig 7). The separation of the six applied laser pulses could be seen clearly, which was in good agreement with similar pulse distances for the experiments (see Fig 2). The simulation in Fig 7 corresponded to regime one, where no creation of step-like structures occurred.

**Fig 8:**
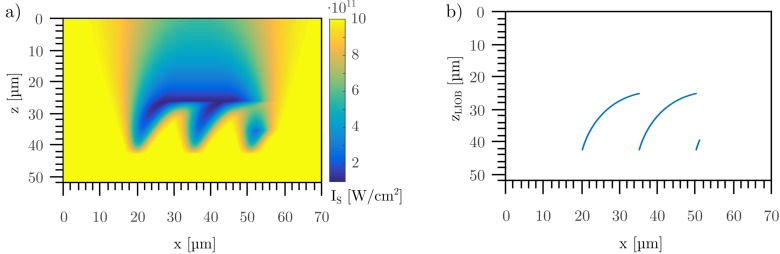
Simulation of the application of 320,000 laser pulses with spatial pulse separation distance sx of 0.1 nm. Shown is the varied threshold Is(x,z) (a) and the position of optical breakdown (b). Due to the laser induced variation of the materials damage threshold, the position of optical breakdown shifts in the direction of the objective lens. This results in step-like structures as can be seen in Figs 1 and 2.

If the lateral pulse distance was reduced, the laser-modified material influenced the position of achieved damage threshold for following laser pulses (see Fig 8). Herein, the cumulative, varied damage threshold of the media caused by 320,000 applied laser pulses with a lateral pulse distance s_**x**_ of 0.1 nm was shown, as well as the position of reached damage threshold for each applied laser pulse. Due to the varying number of applied laser pulses, different color coded scales for the damage threshold in Figs 7 and 8 are applied. With less lateral pulse distance, the cumulative reduced damage threshold of the material leads to an optical breakdown closer to the focusing optic. Hence, the position of optical breakdown is shifted to the direction of the focusing optics. As the position of optical breakdown was further dislocated from the optical beam waist, the reduction of damage threshold due to formula 4 was lowered. Hence, the dislocation of the position of optical breakdown was reduced. This could be derived by Fig 8B: At the beginning, the slope of the positions of optical breakdowns was high, whereas the slope was reduced with increasing applied pulses. This figure looks similar to Fig 1B. After a specific number of pulses, or with the applied parameters in Fig 8 at x = 35 μm, the applied laser intensity at a depth of z = 25 μm did not reached the modified threshold for optical breakdown at that depth. Hence, no additional shift of optical breakdown in the direction of the focusing lens took place. The beam propagated through the medium until the location of z = 42 μm were the threshold was reached at the original focal position of the smallest beam waist. Additional application of further laser pulses led to the already described shift of optical breakdown which could be seen in the creation of step-like structures.

Fig 8 reveals that due to a laser induced reduction in damage threshold, the position of achieved damage threshold moved to the source of the radiation, in this case to the objective lens.

**Fig 9:**
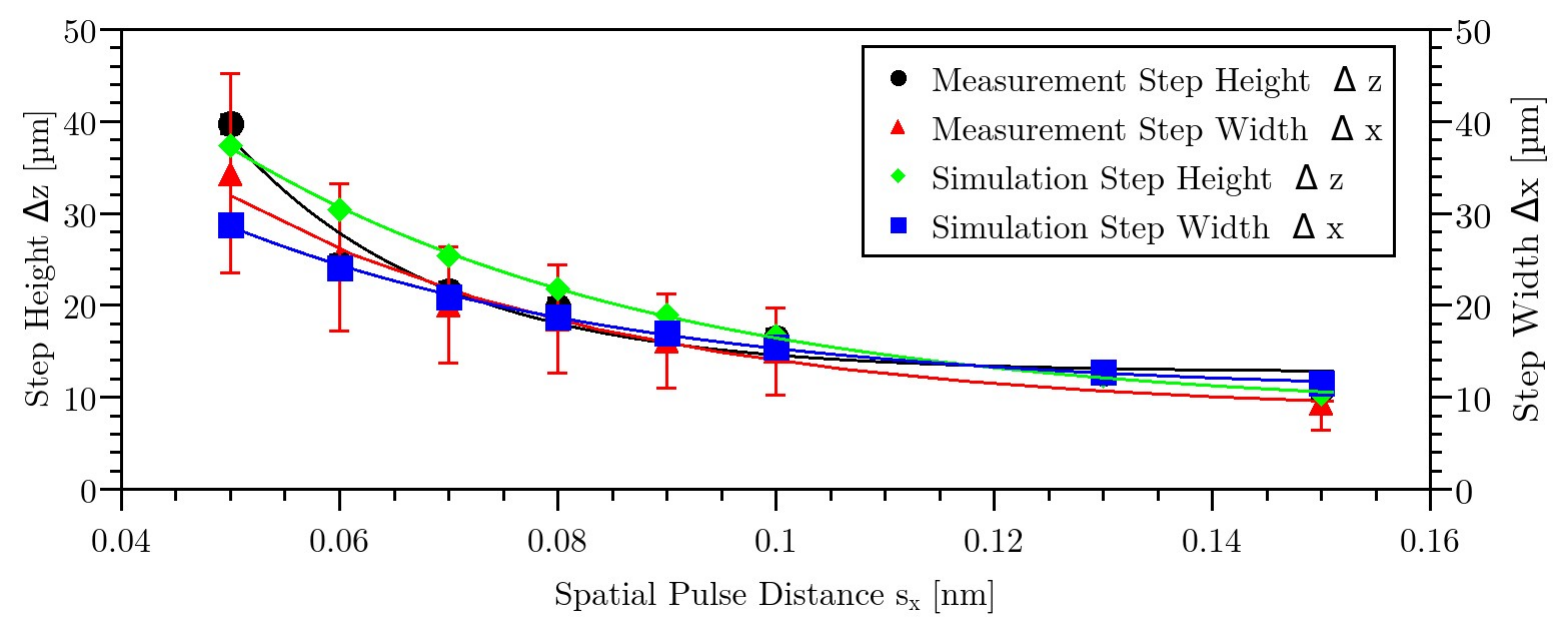
Step height Δz and step width Δx as a function of spatial pulse distance sx as shown in the measurement in Fig 3 and due to the simulation. The error bars derive from the standard deviation over 11 values.

Using the position of the reached damage threshold in Fig 8B resulted in the step height Δz and step width Δx for the simulated values. A comparison (see Fig 9) between calculated and experimentally observed (see Fig 3) values reveals that there is a very good match. Hence, the simulation enables an adequate imaging of the creation of step-like structures in HEMA38 for regime 3. The wave-like structures as observed in regime 2 could not be simulated yet. This might require an adaption of the prefactor in formula 4, which might be a time-dependent variable.

As outlined previously, the step height was independent on the repetition rate (see Fig 5), which implied that the modification of damage threshold is not related to temperature. The absolute modification of damage threshold was low for the simulation. The maximal reduction of damage threshold was only in the order of one magnitude. Hence, this low variation in damage threshold could be caused by chemical modification of the material which was also shown in the Raman-microscopic analysis (see Fig 6). The chemical decomposition could be caused by low-density plasma or color center formation.

The Raman-microscopy showed the creation of an additional peak between 580 nm and 583 nm (1560–1650 rel. cm^-1^) at the laser affected area, which stands for C = C stretching [29, 47]. The laser affected area also showed a broad fluorescence signal, caused by a dissociation of polymers in monomers [29]. Overall, this resulted in a creation of smaller molecules, which could lead to a reduction in damage threshold for optical breakdown.

Astonishingly, an influence on temporal pulse distance was not measured within the range of 10 kHz to 10 MHz. But, as depicted in another study [21], heat accumulating effects were measured above repetition rates of 100 kHz in glass with a thermal expansion coefficient of 8·10^−3^ cm^2^/s [21]. For water, the thermal expansion coefficient is 1.44·10^−3^ cm^2^/s [49] and for hydrogel 1.8·10^−3^ cm^2^/s [50]. Since, the thermal expansion coefficient in glass is 8 times larger than for water, the heat accumulation is not as pronounced in this study as compared to glasses. This could explain that there is no influence of temporal pulse distance on step height measurable (see Fig 5). Another study showed that a temperature increase of 60 K has no influence on damage threshold for water and water-like materials by using ps- and ns-laser pulses [51]. As explanation, the temperature induced excitation of electrons in the conduction band by Boltzmann-distribution were depicted as neglectable [9]. However, at the shortest pulse separation distance of 100 ns, the expansion coefficient of 1.8·10^−3^ cm^2^/s for hydrogels [50] results in a spatial distance of 75 nm. Compared to the diffraction limited spot diameter of 1.6 μm, this is a short distance. Hence, the influence of thermal heat diffusion cannot be completely excluded as reason for the creation of step-like structures.

The used objective lens in the presented study was optimized for a cutting depth of 70 μm thickness after 1.0 mm thick glass object slide. The experimental analysis and the creation of step-like structures was performed at two depths, namely 70 μm and 300 μm. Astonishingly, at such a large depth variation the creation of step-like structures was still achievable, since spherical aberration of such high NA objectives lenses [52, 53] would have caused spreading of the light energy spatially at depths which vary from the designed cutting depth. Hence, the effect of cutting depth on the creation of step-like structures should be investigated furthermore.

## Conclusion

**Fig 10:**
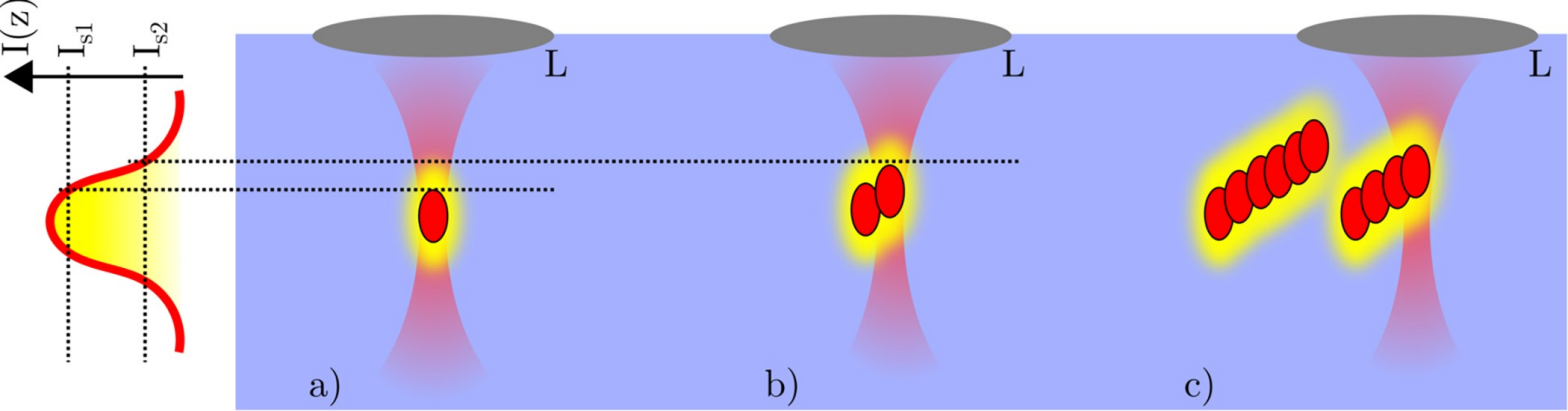
Schematically representation to illustrate the cause of formation of step-like structures inside a transparent hydrogel (blue). Shown is the intensity profile of the laser beam (red) due to focusing through a lens L. a) depicts the optical breakdown (red ellipse) after exceeding the damage threshold. Additionally, a chemical material decomposition (yellow ellipse) due to the laser intensity reduces the materials damage threshold. Hence, b) shows the exceeding of damage threshold at IS2, which leads to a displaced optical breakdown for the second pulse. Since the variation of the materials damage threshold is reduced by increasing distance from the original focus (yellow shading), this results in the step-like structures as shown in c).

The cause of the creation of laser-induced step-like structures in transparent hydrogel was described by experiments and imaged by simulations. Furthermore, parameters were defined, which can enhance or decrease the structuring process. This knowledge can be used for other transparent media, as tissue, glasses, or polymers which are used in ophthalmology or fs-laser-material processing. An adaption can be performed to the work of Ganin *et al*. and Vartapetov *et al*. which used polycarbonate [32, 33]. The formation of step-like structures could be explained as followed (see Fig 10): The first applied laser pulse led to a destruction of the material and additionally to a chemical depolymerization of the surrounding of the destroyed region. This chemical modification caused a reduction in damage threshold of the material, which resulted in a photodisruption of the following applied pulse slightly closer to the focusing objective, compared to the first applied laser pulse. Besides, the second applied laser pulse also changed the chemical modification of the surrounding material. This interaction between laser pulses and the material took place for several pulses. However, the chemical modification was related to the laser intensity, which resulted in a reduced chemical depolymerization distant to the original focal region. Hence, after a specific number of applied pulses, the damage threshold would be reached only at the original focal region (see Fig 10). This creates the step-like structures. The simulated values for step height and step width match the measured ones (see Fig 9). In regard to the assumption of other work [32, 33] a variation in damage threshold leads to the described structures.

Raman-microscopy showed additional stretching of C = C bonding in the laser-structured area, which was not shown in the unmodified area. This indicates that the laser-induced modification of molecule chains leads to the reduction of damage threshold.

The creation of step-like structures due to laterally close applied laser pulses needs to be considered in laser-material processing [2, 15, 16, 17, 20, 22, 23, 24, 25] and laser-medicine [26, 27, 54, 55, 56]. The performed investigations indicate, that the described effect could be suppressed by an increased lateral pulse distance, lower pulse energy or randomized application of laser pulses in the area of interest.
